# Deliberative Lab Communication and the Practice of Ethical Science

**DOI:** 10.1007/s11948-026-00607-x

**Published:** 2026-06-04

**Authors:** Dena K. Plemmons, Kevin M. Esterling, C. K. Gunsalus

**Affiliations:** 1 The Graduate Division, University of California, Riverside, Riverside, USA; 2 Department of Political Science and School of Public Policy, University of California, Riverside, Riverside, USA; 3 National Center for Principled Leadership and Research Ethics, University of Illinois, Urbana-Champaign, USA

**Keywords:** Lab culture, Deliberative communication, Responsible conduct of research

## Abstract

We evaluate an intervention designed to give lab-based research teams an opportunity to intentionally discuss project-related data management practices within their labs, examining how such communication might inform perceptions of the relationship between formal data management plans and lab members’ day-to-day data management practices. In an earlier study, we developed a lab-based intervention that encouraged a deliberative approach to discussions among lab members regarding the practices of data management and authorship, and an exploration of the ethical dimensions of those practices. This present study builds on this prior work, both as partial replication and extension. Here we show the significant effects of the intervention across several dimensions, but importantly and specific to this project, this deliberative communication approach enhances the likelihood that lab members share an understanding of and a commitment to its data management practices, in part because they have been actively involved in shaping those practices. Fostering shared understanding and commitment is crucial for maintaining rigor and responsibility across all aspects of a lab’s work, and is essential for cultivating legitimate, defensible, and ethical approaches to producing scientific knowledge.

The conduct of good and ethical science requires researchers to clearly communicate the reasons for engaging in research practices, justifying the decisions and actions that are made in the research process. When lab members articulate the reasons for their actions, whether inside or outside the lab, they create opportunities for those reasons to be critically examined. Those reasons that survive scrutiny serve as the basis for reason-based collaboration in the lab’s work.

This kind of foundational communication, grounded in intentional discussions about the assumptions, processes, and ethical dimensions of the day-to-day work being done in the lab, is more than routine conversation. Effective, deliberative communication is structured, inclusive dialogue oriented toward reason-giving, mutual accountability, and the justification of shared decisions and practices (Habermas, 1984; Gastil, 2006; Englund, 2006; Stahl, 2025). Deliberative communication in the lab enables lab members to make collective decisions that members, the discipline, and the broader public perceive as valid and legitimate, an outcome that requires ongoing reflection on the lab’s internal standards and practices, and provides opportunities for sustained, purposeful attention to the ethical dimensions of its work.

These lab-based practices, along with the communication that shapes and supports them, occur within a broader landscape of policies, laws, and regulations governing scientific activity. Yet formal policies alone neither define how a lab functions nor ensure that its practices are grounded in deliberation and reasoned exchange. In reality, the relationship between policy and practice is complex and variable: sometimes regulation is translated into specific, purposeful action; other times, practice reflects little more than unexamined habits and routines; and often, it is some combination of both. Daily lab work is also shaped by the immediate, project-specific demands of research, which can pull practice further from formal policy. This is especially true when policies originate from external mandates, such as RCR training requirements or the data management plan requirements imposed by federal funders, with a resulting focus on compliance displacing genuine engagement with how those obligations relate to actual lab work.

Most current approaches to RCR training (typically, though not always, online asynchronous modules or classroom instruction removed from the lab context, and primarily directed at trainees) satisfy the minimum threshold of compliance rather than responding to the *intention* of the requirement, adopting approaches that are expedient and scalable over those that are most effective, relevant, and meaningful. As a result, in these typical approaches, there is little opportunity for laboratory members to develop the interpersonal competencies and capacity for reasoned dialogue needed to recognize ethical issues as they arise and respond to them thoughtfully in the course of their work.

For data management in particular, such an externally driven formal plan frequently exists alongside, but often only tenuously connected to, on-the-ground research practices (Van Loon et al., 2017; Williams et al., 2017; Smale et al., 2020). Deliberative lab-based discussions can help uncover this disconnect and allow for a lab’s collective examination of the relationship between the lab’s formalized, pre-existing data management plan, and the actual practices that emerge in response to the evolving, complex, project-specific demands of their work.

In a previous study (Plemmons et al., 2020), two of the authors (Plemmons and Esterling) led a team that developed a deliberative communication intervention called “Institutional Reengineering of Ethical Discourse in STEM,” or iREDS, a randomized control field trial that encouraged a deliberative approach to lab-based discussions among lab members regarding the practices of data management and authorship, and an exploration of the ethical dimensions of those practices. This previous study found that the intervention strengthened within-lab deliberative communication in many ways and helped lab members understand the importance of the consideration of ethical practice. However, in this previous study we did not evaluate whether the improved discourse was linked to changes in research *practices*. Specifically, we did not explore how or if this approach affected understandings of data management practices in particular, nor did we examine perceptions of how, if at all, the data management plan was seen to be aligned with practices of data management in the lab.

The present study, entitled “Deliberative Lab Communication” (DLC), builds on the iREDS project with two related goals. First, we aimed to replicate findings from the earlier study to demonstrate the effectiveness and generalizability of this approach at a different university, and across the same dimensions, including laboratory climate and the cultivation of deliberative communication ideals. Second, we sought to extend this previous work by examining how such intentional, deliberative communication might shape lab members’ understanding and perception of the relationship between formal data management plans and the everyday research practices of the lab. This paper reports the results of this DLC intervention.

## Methods

All materials and methods were reviewed and approved by the Institutional Review Board of the University of California, Riverside (UCR 22089) and the Institutional Review Board of the University of Illinois, Urbana Champaign (UIUC IRB24-0808). We obtained voluntary informed consent from all participants before they took part in the studies.

### Participants

The DLC research was conducted at the University of Illinois in active faculty-led laboratories across a range of STEM fields.^1^ Individual email invitations were sent on our behalf by the Office of the Vice Chancellor for Research and Innovation to 968^2^ principal investigators conducting federally funded, lab-based research; one communication went out at the beginning of the Fall semester of 2023, another at the beginning of the Spring semester of 2024. The email was an invitation to meet with this project’s PI (Plemmons) to discuss the project. If there was interest, then the consent process took place. Upon obtaining consent from a principal investigator, we were then able to seek consent from each lab member; lab members included postdoctoral researchers, graduate students, project/research scientists, support staff, and undergraduate research assistants. As the lab was the primary unit of analysis, a laboratory qualified for enrollment into the study if at least 50% plus one of its members provided consent. Only consenting lab members participated in the surveys, although nonconsenting members could still attend the facilitated discussion.

51 PIs (5%) initially indicated an interest in meeting to discuss the project, and 36 PIs (70%) subsequently enrolled in the study^3^; of those 36 PI enrollments, 19 (53%) labs eventually met our eligibility criteria and completed the study, with 16 failing to meet the eligibility criteria, and with one PI deciding not to continue after signing the consent.

Our final sample consisted of 107 members of 19 research laboratories and included principal investigators (20),^4^ graduate students (52), postdoc researchers (12), undergraduate research assistants (10), research scientists, (9) and support staff (1); 3 were categorized as other^5^.

There were 8 university departments/programs/centers represented among the respondents; those were Agriculture, Consumer, & Environmental Sciences; Grainger Engineering; Liberal Arts & Sciences; Applied Health Sciences; Medicine; Veterinary Medicine; the Vice Chancellor for Research and Innovation; and the Office of the Vice Chancellor for Research and Innovation Institutes. Within these eight, there were approximately 27 disciplines, ranging from (not exhaustive) Food Science and Nutrition to Evolution Ecology Behaviour to Information Sciences to the Prairie Research Institute.

In our sample of participants, 44 (41%) identified as male, 4 (4%) self-identified as African American, 30 (28%) self-identified as Asian, 18 (17%) self-identified as Hispanic, 61 (57%) self-identified as white, and 10 (9%) chose not to any to answer any of the demographic questions.

The procedures in this study – pre-survey administration, facilitated discussion intervention, and post-survey administration – were similar to those in the prior study on which this project is based (Plemmons et al., 2020). However, in this current study, the structure, content and delivery of the intervention material were slightly different, there were no peer mentors, we did not make use of any data management platform, and there was no randomization into treatment and control groups, though as shown in the data analysis below, we make use of the iREDS randomized control units as a natural experimental control group for this study.

### Survey Administration

The pre- and post-surveys were administered online using the Qualtrics survey platform.

The pre-survey was delivered to participants between two and three weeks before the facilitated discussion, and the post-survey was administered ~ 4 months after the discussion. The pre- and post-surveys were identical, and each had a total of 89 items, measuring aspects of participants’ personal beliefs and opinions about ethical research practices and their perceptions of climate and communication within the lab, as well as understanding of and experiences with laboratory practices related to data management. There was also a question asking about any other data management discussions they may have been involved with, both prior to and during the months of the research, to help identify any confounding data management interventions.

22 participants took part in the first but not the second assessment (i.e., 4 months later); 6 participants took part in the second assessment but not the first; and 79 participants completed both assessments.^6^ Of the singles, 2 are PIs, and 26 are laboratory members. We retain all observations in the analysis. Our procedures for handling the missingness in our sample are discussed in the methods appendix.

### Intervention

The laboratories enrolled in the study participated in a ~ 90 min facilitated, loosely structured discussion^7^ about data management, however conceived and articulated in that lab. The discussion, among each lab’s members, focused on current projects in their lab. The intervention began with a brief definition of deliberative communication, and then a quick orientation to “data management” very broadly defined (the training material used for the research is available at https://osf.io/b6rm2/overview). After these brief orienting remarks, the conversation was opened to the lab with a suggestion that the lab members think about the kinds of data generated in the lab, and the issues they find themselves most attentive to in managing those data. Importantly, lab members were made aware that the facilitator was not a data management expert – indeed, was not trained in their discipline – and was not there to evaluate the lab’s data management practices, nor would they be offering resources, strategies, or suggestions about the lab’s data management. After that first ~ 15 min introduction, the remainder of the conversation was led almost entirely by the lab members, with occasional questions as follow up, clarification, or challenge from the facilitator.

### Data Management Plans

We requested the written data management plans/policies of the participating labs both before and after the intervention to assess any changes.

**Fig. 1:**
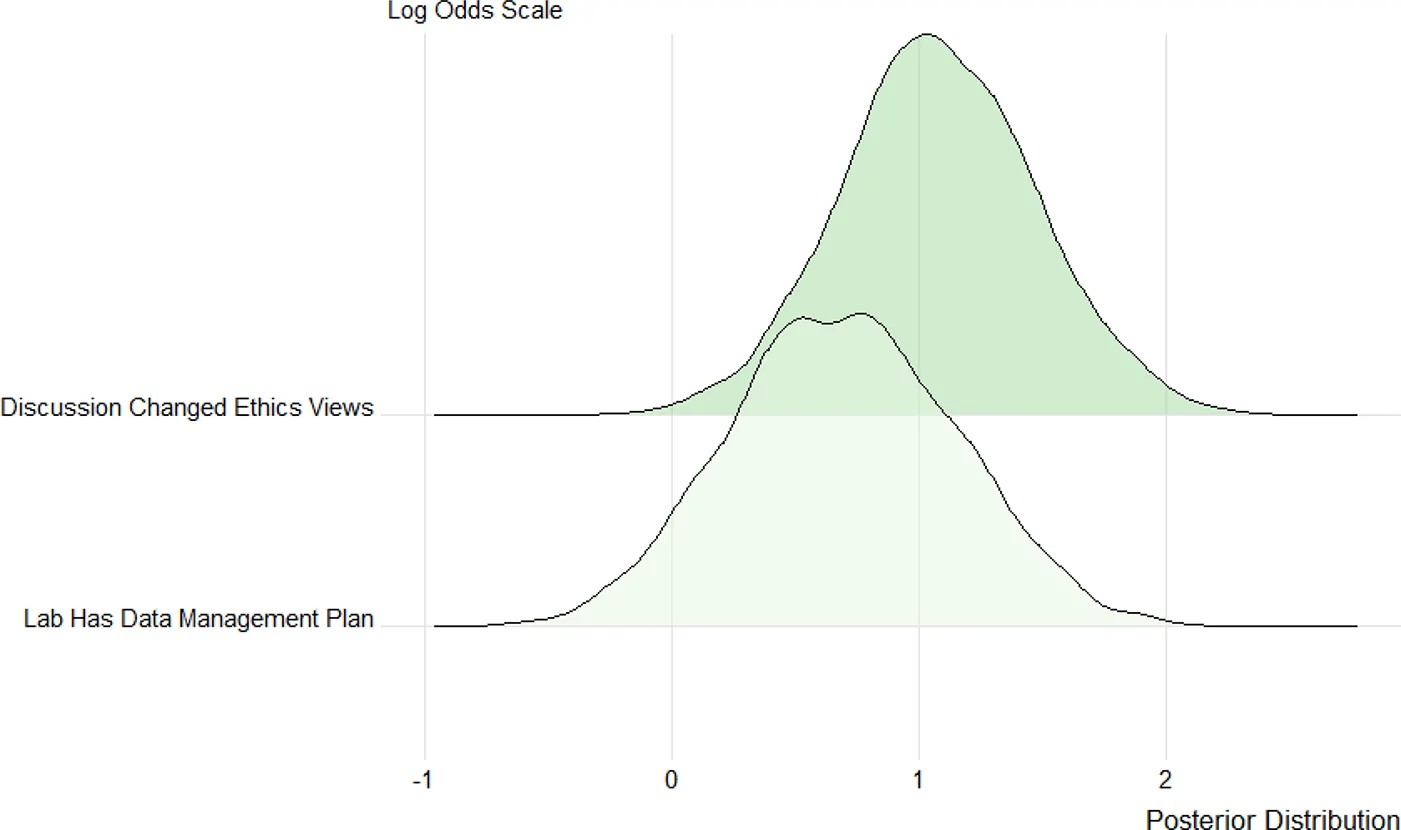
Descriptive estimates. Bayesian posterior distributions for within-subjects pre-post changes in log odds estimates, allowing for within-lab survey response dependence. Bottom Row - “Lab has data management plan,” logit likelihood, and Top Row - “Discussion changed ethics views,” ordered logit likelihood. *N* = 107. See the methods for dependence structure, prior distributions and missing data imputation

### Causal Effect Identification

To identify the effects of the intervention using observational data, we make use of two designs. The first design relies on a within-subjects comparison of post-test response distributions to pre-test response distributions. The second design is difference-in-differences (DID), which compares the changes in the treated group to the corresponding changes in the iREDS randomized control group that was not exposed to the intervention. We discuss the assumptions required to identify causal effects under each design below in the results section.

## Results

The pre- and post-surveys consisted of item-specific Likert-response items assessing attitudes and behaviors relating to lab climate, communication, and data management understanding and practice. We reproduce the survey items and response categories in the SI. Our outcomes of primary interest for the causal analysis were latent scales that we measured using sets of survey items that we describe below. Two individual items, however, were of descriptive interest as they measured exposure to the intervention: These were: “Does your lab have a data management plan/policy?” and “Have you changed your views about ethical research practices based on discussion within your lab?” We used Bayesian nonlinear regression models to estimate the within-subject descriptive pre-post change for these two individual survey items.

Figure 1 gives the posterior distributions for the descriptive estimates in log-odds for pre-post changes in the individual survey items. The figure is a pair of marginal density plots of the posterior distribution of the estimate. We found a significant difference for the item asking if the respondent’s views on ethics changed as a result of discussion in the lab, with 99% of the posterior distribution to the right of zero. We also found a significant difference for the item asking the respondent to report the presence of a lab data management policy, with 93% of the posterior distribution to the right of zero. This result does not necessarily mean that labs created new data management plans as a result of the intervention, and indeed we report below the opposite; instead, the intervention may have raised awareness of the existence of a data management plan.

The survey also had batteries of questions that measured six latent dimensions using scaling techniques, and we used these scales as our primary outcomes for the causal analysis. The first scale measured the respondent’s beliefs about the relevance of ethics discourse for their research and work. The items that loaded on the *Relevance of Ethics Discours*e scale were: “How relevant to your area of work is learning about ethical research practices?” “To what extent do you agree that seeking others in your department to discuss ethical research practice is your responsibility as a scientist?” “To what extent do you agree that seeking out others in your lab to discuss ethical research practices is your responsibility as a scientist?” “Do you have discussions with members of other labs regarding ethical research practices?”

The second scale measured the extent to which the participants perceived the discussion in their lab to be reason-giving, respectful and equal, which are core requirements in the concept of deliberation. The items on the *Respectful Discussion* scale were “If there was a disagreement about research practices, would lab members in your lab make reasonable points and try to make valid arguments?” “When discussing research with your lab, does everyone have a real opportunity to speak with no one inappropriately dominating the discussions?” “When discussing research with your lab, do lab members listen to one another respectfully and courteously?” “When discussing research with your lab, do lab members seem to hear and understand your views?” “How confident are you that your lab co-workers will use ethical research practices when conducting research?”

The third scale measured climate and the amount of disagreement in the lab, which is a measure of the constructiveness of within-lab communication. The items on the *Lab Disagreement* scale were: “How often are there disagreements in your lab about research practices?” “How often has the data management plan/policy resolved disagreements in your lab?” “How often are there disagreements in your lab about data management?”

The fourth scale measured the respondent’s self-reported understanding of the reasons and rationales for having a data management plan. The *Reasons for Data Management Policy* scale items were: “Do you understand the rationale for having a data management plan/policy in your lab?” “Do you understand the importance of having a data management plan/policy in your lab?” “Do you understand the implications for having a data management plan/policy in your lab?” “How comfortable do you feel challenging the data management plan/policy?”

The fifth scale measured the respondent’s perception of the importance of open science practices with respect to the preservation of replication materials, which is a key feature of good data management practice. The *Preserve Replication Materials* scale items were: “How important to your lab is archiving records of earlier versions of data sets?” “How important to your lab is archiving records of earlier versions of manuscript drafts?” “How important to your lab is archiving records of earlier versions of lab materials?” “How worthwhile is it to maintain electronic copies of lab materials and methods?”

Finally, the sixth scale measured the respondent’s perception of how the data management plan works in the practice of research in the lab. This is a new scale that was not included in the original iREDS analysis.^8^ The *Data Management Plan in Practice* scale items were: “Do you know how the data management plan/policy works in practice?” “Have you seen the data management plan/policy put into practice?” “To what extent do you understand how to implement the data management plan/policy in your own work?” “How compelled do you feel to follow the data management plan/policy?” Importantly, all of the items for this sixth scale were only administered if the participant responded yes to the question asking if they knew their lab had a data management plan. As a result, this scale was only estimated for this subset of respondents (*N* = 60 on the pre-test and *N* = 59 on the post-test).

To identify the causal effects of the intervention using observational data, we made use of two designs. The first design relies on a within-subjects comparison of post-test response distributions to pre-test response distributions. The within-subjects design identifies causal effects under the strong assumption that there was no confounding during the study period. Unlike the iREDS study, the DLC study did not randomize labs into treatment and control conditions, and so the second design is difference-in-differences (DID), which compares the changes in the treated group to the corresponding changes in a control group (from the iREDS study) that was not exposed to the intervention. DID identifies causal effects from observational data under a weaker “parallel path” assumption, that the changes in the treatment group outcomes would have run in parallel to those observed in the control group, had the intervention not occurred (Wing et al., 2018). The DID relies on weaker assumptions to identify causal effects compared to the within subjects design in that it accounts for confounds that are experienced by both treatment and control groups in a natural experimental design.

While the within-subjects design relies on strong assumptions, an assumption of no confounding during the intervention period was reasonable in this study given that our survey found no evidence that the participants received alternative data management interventions during the study period. However, two confounds remain that are integrated with the study design. First, we know that during the study period, DLC participants were administered the pre- and post-surveys, and it is possible that responses to the second survey reflect a retest bias. Second, we know that time elapsed between the pre- and post-survey, and it’s possible that responses simply drift systematically over time. To address these known confounds, and to weaken the no confounding assumption, we used difference-in-differences making use of the control subjects from the previous study (Plemmons et al., 2020), who were administered the near identical questions that we used for this analysis and experienced a similar survey administration timeline. The DID using the iREDS controls was able to eliminate the two known confounds of survey administration and time. However, using the iREDS controls does not eliminate any exogenous event confounds that happened during the DLC study period and hence strengthens the parallel path assumption. Below we show the DID results match the RCT benchmarks from the prior study, while the within-subjects results have similar point estimates, but higher precision compared to the RCT.

For estimation, we used computational Bayesian MCMC inference. In all analyses, we used a multilevel model that accounts for response dependence due to communication within labs. We explain the estimation procedures in more detail in the Methods.

**Fig. 2:**
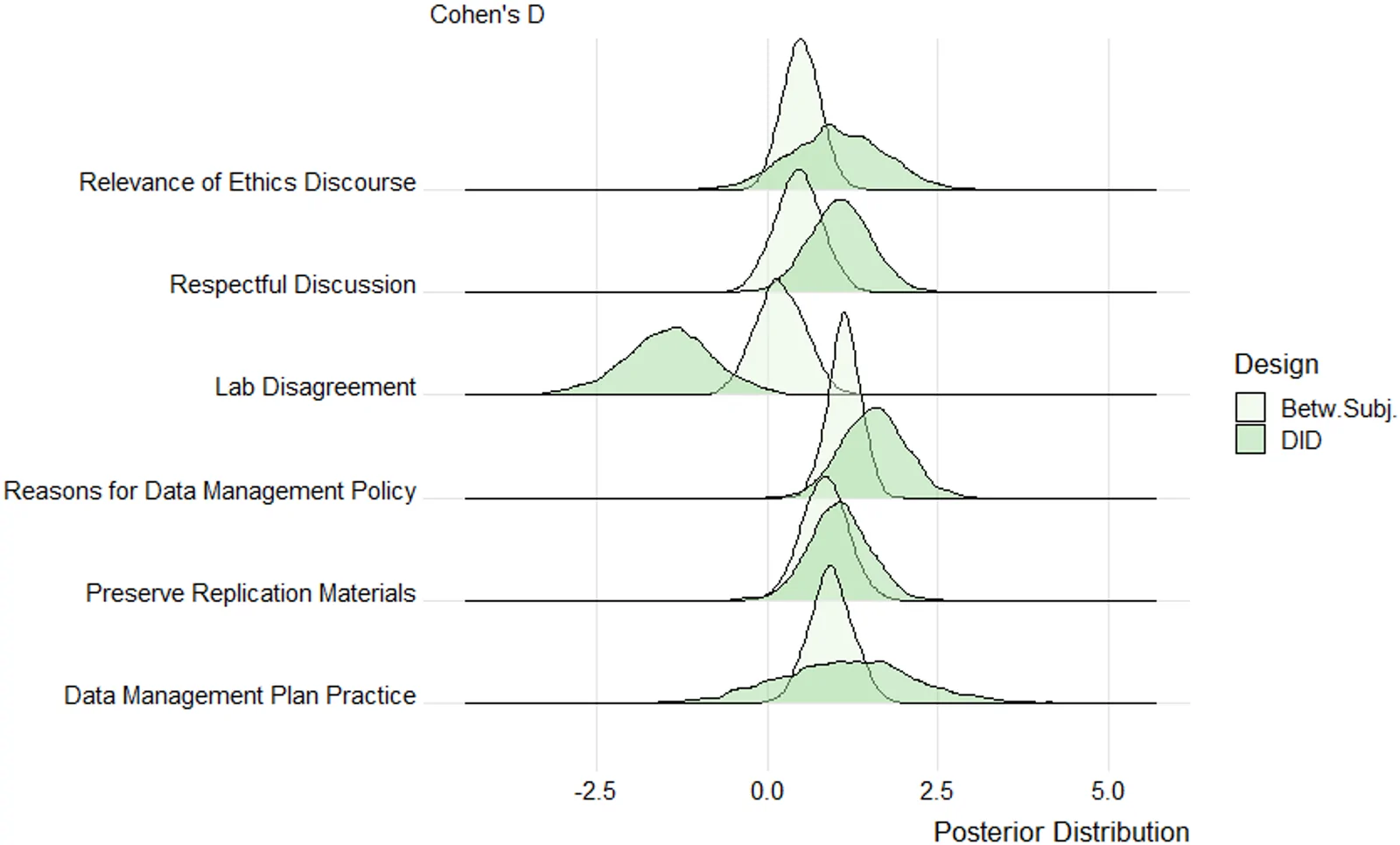
Causal effect estimates for the outcome scales. Bayesian posterior distributions of Cohen’s D causal effect estimates, allowing for within-lab survey response dependence. Light Shading - Between-subjects design (*N* = 107). Dark Shading - Difference-in-Differences (DID) design (*N* = 196). See the methods for implementation of the scale models, dependence structure, prior distributions, and missing data imputation

Here we report the causal effect estimates for the scale outcomes in Fig. 2. The figure shows the posterior distributions of the Cohen’s D marginal effect of the treatment for each of our six scales in a density plot, for each of our two designs. Lighter fill indicates the between subjects estimates and darker fill indicates the DID estimates. The DID estimates in the top five scales (i.e., excluding the Data Management Plan Practice scale) are nearly exact replications of the RCT benchmark findings in the original iREDS study, while the between subjects estimates have similar point estimates but much lower variances. As a result, we take the DID estimates as preferred. Note that the *Data Management Plan Practice* scale has a flatter posterior in the DID estimates, which is expected since the number of non-missing observations available for this analysis is much lower than the other scales.

The results for those individuals who participated in the DLC intervention were similar for those who participated in the prior iREDS research – lab members were more likely to regard ethics communication as relevant to their scientific work (Cohen’s D = 1.02), to characterize discourse within their labs as respectful and open to diverse perspectives (Cohen’s D = 1.07), and to report a more constructive discussion climate, indicated by reduced disagreements over scientific practices (Cohen’s D = -1.45). These three scales suggest that labs participating in the intervention more closely aligned with deliberative communication ideals, perceiving discussions of research and ethical practices as more respectful, open, relevant, and constructive. Those who participated in the intervention also were more likely to understand the importance of and rationale for having a data management plan (Cohen’s D = 1.57), and report that the lab is more likely to archive and preserve replication materials that underlie the research (Cohen’s D = 1.05). These results show a nearly exact replication of the original iREDS findings, which is notable in that they show the generalizability and external validity of the original study findings (Plemmons et al., 2020) across time and across universities. And in each case, the posterior distribution is mostly either to the right or to the left of zero, which in a Bayesian framework implies there is a very low probability that the treatment effect is zero.

The data collected in the original iREDS study regarding the items in what is the sixth scale in this current study did not have a large enough sample size, as explained above, to evaluate the impact of the intervention on the respondent’s understanding of the lab’s data management plan and its relationship to lab practices, how to implement the plan in their own work, and the extent to which they feel committed to following the plan. Here, the DID result indicates a significant improvement in practice along these lines (Cohen’s D = 1.15), although only 87% of the posterior is to the right of zero. The relatively higher uncertainty for this item is to be expected however since the items were only administered to the subset of respondents who were aware of the lab’s data management plan in the first place, so the number of non-missing observations is much smaller.

In addition to the survey, we also requested the data management plans, both before and after the intervention, from those labs enrolled in the study to assess whether the deliberative conversation resulted in any changes to the written plan.

We collected 14 documents from the 19 labs prior to the facilitated discussion. Of the remaining 5 labs, 4 of the PIs stated they had no plan, and one PI mistakenly wasn’t asked for their plan pre-intervention (and so was not included in this part of the analyses).

Post-intervention, we received documents from 13 labs. Five PIs did not provide a plan post-intervention; of those, two had provided a document pre-intervention but did not provide one post-intervention. One PI who did not provide a document pre-intervention, though was asked for one, did provide one post intervention.

These documents (variably referred to by the labs themselves as a data management plan, a lab manual, study monitoring procedures, a lab expectations document) were received **both** pre- and post- intervention from 12 labs. For those 12 labs, some responses to our request for the data management plan pre-intervention included: “I have to admit that we have not been very good at putting a formal data management plan into action for our group, but we have had sporadic discussions on the topic, which to some extent are documented in our group meeting agendas. I guess the discussion on Friday may prompt us to do better than that” to “we do not have a formal one, I have attached a plan submitted for a grant proposal that aligns with our general policies” to “the file I’ve attached is the best that we have. We hope this helps” to “We do not have a formal data management plan, but probably should have one.”

Responses to our request post intervention included: “The data management plan has not changed since we provided it last year” to “Here is our current data management plan. I don’t think we altered it after our meetings with you all unfortunately” to “I don’t have anything other than the document that I sent to you previously from my NIH grant” to “we don’t have a formal plan written out yet. We hope to do it, but have not had time to finalize it” to “What do you want me to do if there are elements of our data management plan that are not written down?”

9 of those 12 labs^9^ (75%) had made no changes to the documents or the relevant sections of documents shared at the pre-intervention time point. For the remaining 3 labs, we did find the documents were different at the two collection points, though not substantively so for 2 of those labs. One lab added a section to include policies for access and sharing and for addressing data protection and privacy concerns to an otherwise unchanged document; one lab added a different structure and made two simple additions regarding where not to store data, and what to do with data/lab notebooks when leaving the lab. Finally, while one of the labs had submitted, pre-intervention, the results from th a fairly standard data management plan, addressing areas such as the appropriate processing of samples, how and where to record measurements, sample and file naming conventions, and integration of data across multiple repositories, the post-intervention submission came with this explanation and a new document: “I do not have a written data plan, but here is a quick overview of how I see the world in my lab”.

As part of a separate and planned evaluation of this project, collaborating colleagues conducted follow up interviews with some of the research participants. Those responses helped us to more fully contextualize some of the survey results and further explore those results in light of the outcomes from collecting the data management plans. Follow up interviews were conducted between 9 and 16 months after the post-survey administration (13–20 months after the intervention). 54 members from 12 labs^10^ were invited to participate, with 9 lab members (16%) consenting.^11^ The respondents were PIs (3), graduate students (3), a post-doctoral scholar, an assistant research scientist, and a lab manager.

The semi-structured interviews (the protocol can be found at https://osf.io/b6rm2/overview) were designed to explore perceived lasting changes in approaches to communication since the intervention, increased frequency of discussions which included the ethical dimensions of lab practices, and perceived changes in efforts to be more intentionally inclusive in lab conversations. While the small n limits generalizability, we were able to preliminarily explore durability beyond the 4-month post-test across a few different dimensions.

A PI was asked

…since the DLC intervention, have you noticed people in your lab more frequently raising questions about lab practices in the context of conducting research?

A: I would say yes, particularly when it comes to… the ways in which we collect our data, so we have much better communications and systems about signing up for shared equipment and where we’re storing lab data on those pieces of equipment so that people have access to it and understand what was already done versus what’s not been done, if that makes sense.

Q: Were there any ethical dimensions to these conversations?

A: Not in the context of somebody … using that word ethics or not ethics, but around maybe when we’re looking at our data and seeing variability in some of the outcomes, like the ethics behind needing to show all the data points and needing to design the next set of experiments to try to minimize some of that variability. So more ethics on reproducibility and reporting as opposed to the way… in which we write down data. So more along the ethics related to what the data are telling us as far as the conclusions we can make and the repeatability.

An assistant research scientist responded to the question “…did this intervention help you in having conversation in your lab?” with

…I think maybe it broke some of the ice of acknowledging that even the higher ups, there are things that we have blind spots on or we need to ask questions to. And so it kind of opened up and warmed up the playing field for everyone from the boss to the new undergrad. So I think that kind of helped set a baseline of no, yeah, we actually, we all have questions and there are no bad questions, and it’s better to ask the question than not know….

A graduate student responded to the question “… would you have paid attention… as intentionally (to) the inclusion of voices, inviting diversity of viewpoint and experiences, if your lab had not participated in this DLC intervention?” with

No, I don’t think so. And I think that it’s just one of these things that you don’t necessarily think about unless it’s brought up. Even if you’re in practice doing, I don’t think that any of us were like, oh, we need to make sure that everyone’s included, so we’re going to dedicate this time for everyone to speak… once you talk about it, you’re like, okay, yeah, I mean, I support this and I think everyone in the lab does. That makes sense. But it’s not something that you necessarily think about unless it’s brought up to you and said specifically, this is a good thing for you guys to do, then it makes sense and then you think about it.

One focus of these interviews was the topic of data management, inviting respondents to reflect on the relationship between a lab’s data management plan and its day to day practices, with specific reference to the survey’s quantitative findings on the sixth scale, and soliciting feedback about / drawing attention to the fact that in most cases, a lab’s data management plan did not change after the intervention. Several of these responses offered valuable insights on the ways in which data management plans and practices are characterized; how they might function in parallel; and the potential for each to influence the other.

Respondents variously described data management plans as idealized, estimated, speculative, required, standardized, or broad, while practice was characterized as what happens on the ground, in response to the unpredictability of the research process, often in chaos or stress, certainly in the midst of extreme time pressure and limited bandwidth, and all the while different people rotating in, bringing in new perspectives and expectations, and out, taking with them a kind of institutional knowledge about managing data in that lab. These realities influence whether, and how, plans and practices aligned.

A couple of responses to the question about the plan/practice relationship noted that while the data management plan might indeed be good and valuable, *adherence* to that plan had ebbed, for all the reasons noted above (time pressure, new people coming into a lab, seasoned students leaving, limited bandwidth) but also because the importance of the connection between plan and practice hadn’t really been made or become clear, and there was nothing intrinsic to the plan per se that prompted adherence; one graduate student said

It’s not that the plan was bad, it’s that we didn’t really all agree that we needed to follow it, if that makes sense. But once we talked about it more, we’re like, okay, this is actually important and it’s not that hard for us to do, so we’re just going to do it right.

A lab manager responded this way:

I think probably some of the discrepancy would be maybe it wasn’t talked about or there’s ‘here, just follow this step by step for data management plan’. We don’t really know why we’re doing it, we’re just doing it. And I think maybe the perception changed because this intervention forced you to think about, okay, why are we doing it? What is beneficial from this?

To the question “…given that there were no observed differences between the data management plans from the pre-post surveys, first question is what do you think about this? Any thoughts about this possible discrepancy?”, a postdoc responded:

It doesn’t really surprise me to be honest. And I think that for me has something to do with how abstract a plan (is)…. To me, it doesn’t carry any, like I said, it doesn’t carry an inherent or intrinsic motivation to it.

Importantly, several responses acknowledged that the DLC intervention was helpful in making that connection actionable.

Other responses focused on the ways practice could and should shape a plan, most notably, that at a minimum, the plan should reflect actual practice so that it might reliably and constructively guide future practice; as one respondent noted

I think a plan as such would only be viable if you take into account the practices of it. And I think in order to do that, a group would need to gather and have such discussions like we had… every half a year, every year, and take into account feedback from the practical side into the plan.

A PI responded:

I mean, I think it’s always good to have a plan, but then real life also interferes, right? So I think you want to make sure there’s always enough flexibility built in anyhow. [Similar to how] we do our experiments… maybe we encounter things that actually go different than what we expected. then we’ll have to revisit it and maybe adapt our plans for the future. And I think data management plans are not any different in that respect.

A postdoc responded:

A data management plan to me is like I said it’s a document that writes out how data is supposed to be taken and stored and where it should go. It is almost a list of commandments about data handling, and acquisition, whereas a data management practice is what people do and remember when they’re in a lab.

Finally, some participants questioned whether the written plan itself was or even should be the central element in data management; this was a not uncommon response across the interviews, with the respondents drawing a distinction between the plan on paper and the *doing* of data management; as one PI reflected:

I wonder how important is the written document? If you are doing the practices and you have the task that people are doing and they have guidance on what they’re supposed to do, but it may be separate from what’s in the data management plan. I guess I wonder where should the emphasis be, if we want people to constantly be making sure their data management plan is updated? Is that really the most important thing? I don’t know.

Another PI noted:

I think actually what we did instead is have this as more of a regular communication and set up (a) living kind of document – spreadsheets that people log into every time they’re doing an experiment as opposed to a protocol for doing it. You know what I mean? I think we just implemented doing these things instead of writing a plan for doing that.

## Discussion

In this paper, we have presented results showing the effectiveness of this Deliberative Lab Communication approach in engaging all members of a lab, including the PI, in conversation about practices in which the lab engages. In both this and our prior project, we were exploring two lines of inquiry. At a topical level, we examined how labs understand and navigate their data management practices (and authorship practices in the iREDS project) as articulated during the intervention, and in relation to specific research projects, along with lab members’ perceptions and understanding of those practices as reported on the survey. At a broader level, we were asking fundamental questions about lab communication and culture.

Similar to the findings from the iREDS project, our results demonstrate significant and lasting effects of the intervention across multiple dimensions aligned with the benefits of deliberative communication, including improvements in laboratory climate, and increased awareness of the importance of lab-based discourse on research ethics and its relevance and underlying rationale for their work. Importantly, and specific to our exploration of how the enrolled labs conceptualize data management, we found that the DLC framing increases the likelihood that all lab members share both an understanding of and a commitment to their lab’s data management practices. The follow up interviews with lab members reinforced and gave some context to those survey findings regarding the lab members’ assessments of their lab’s data management plan and practices (scale 6): the intervention made participants more aware of both the gap and the resonance between plans and actual practice, prompting consideration of adjustments to bring the two into closer alignment when necessary. The respondents noted that, in some cases, these practices are guided by a formal data management plan, while in others, strong practices emerge even in the absence of documentation in that specific form.^12^

The interviews showed that in those cases where there was no explicit written formal plan, specific practices were still yet aligned with the lab’s projects and the broader goals of the research, and despite no *formal* documentation in every instance, guidance was nevertheless embedded in lab manuals, research team meeting notes, procedures documents, communicated through informal conversations between members, or otherwise integrated into the projects themselves and into the routines of the lab. The lab members perceived that the expectations for data management were addressed in many other kinds of documents and/or activities than just formal data management plans; there was an understanding that each lab did indeed have plans for managing its data, whether or not those aligned with the formal “data management plan” submitted in fulfillment of funder or other regulatory requirements (see for example Williams et al., 2017), contextualizing the management of data in actual practice, such as collection and analyses, and in concerns of reproducibility.

The results suggest that deliberative lab-based discussions can clarify an important point: while a formal data management plan is necessary and beneficial for many reasons, it alone may not ensure responsible data management. Good and responsible practices evolve through ongoing deliberation, adaptation to specific projects, and responsiveness to the complexities and unexpected challenges of research. Ideally, such practices are incorporated into an existing formal plan, but that is not always the case, and even in those instances when the plan is only tenuously connected to practice, good and responsible data management can and does still happen, in part because lab members have been actively involved in shaping those practices.

In addition to these new findings specific to data management practices, this study replicated results from the prior study (Plemmons et al., 2020), showing that the deliberative communication approach helps orient lab members toward responsible and ethical practice, reinforcing its value as a particularly effective method of education in the responsible conduct of research.

If we view one of the primary goals of RCR and research ethics education as fostering and sustaining a community of practice, it follows that the research environment itself should be a key site for this work. It is in research settings that individuals learn their science, engage in collaborative and social interactions around that science, and encounter, often only implicitly, the ethical dimensions of research practice. As the results show, this DLC approach helps make those ethical dimensions explicit and ties them to the actual work of the lab.

Responsible practice, in the context of scientific research, necessitates that all members of the laboratory engage in reason-giving and evaluative judgment explicitly situated within the concrete circumstances of their work. In this situated practice, the epistemic (“knowing”) and the practical (“doing”) are inextricably linked. Regularly integrating discussions of ethical practice into the day-to-day work of the lab ensures that ethical considerations are routinely identified and articulated, relevant and actionable, reinforcing ethics as integral – *foundational* – to good scientific work, rather than something which is separate from practice (Ingold, 1993; Lave, 1997; Whitbeck, 2001; Fryer-Edwards, 2002: Peiffer et al., 2008; Plemmons & Kalichman, 2013; Kalichman, 2014; Plemmons & Kalichman, 2018). Indeed, this kind of ethical reflection then becomes part of the practice itself, sustained through on-going deliberative communication.

Integrating deliberation in routine lab interactions, in turn, offers the potential for it to become an enduring feature of lab culture; as John Gastil (2006) wrote

Fortunately it is likely that deliberation is a self-reinforcing process. The more often we deliberate together, the better we become at it, the more we come to expect it, the more often we expect it to work, and the more motivated we are to try it.

We can assume that the effects shown in this research *will* fade if the lab doesn’t continue with this approach to within lab communication, but we suspect that many labs will see the value in this approach to addressing the many practices, not just data management, in which the lab engages to produce knowledge, and which could benefit from a reasoned, collective, and deliberative lab wide conversation.

Importantly and distinctly, these deliberative lab discussions include the PI, an inclusion which tends not to be part of typical approaches to education and training in the responsible conduct of research. Many such approaches focus almost exclusively on the individual (usually, the graduate student or “trainee”) and are often limited to knowledge-transfer exercises completed in isolation, not considering the actual work being done in the lab, nor including those with whom one enters into and navigates that work. Including the PI in these discussions serves to reinforce that the responsibility for ethical research practices, and consideration of the same, is a *shared* responsibility (National Research Council, Institute of Medicine, 2002; Pimple, 2013; National Academies, 2017; Philips et al., 2018).

As in the conduct of ethical science more broadly, and in specific responsible research practices, the way norms and principles are established and communicated has a significant impact on how well, or even whether, they are followed. Developing these norms collaboratively, through a deliberative process of critical reflection and meaningful communal work, along with the formal policies and informal practices to support them, helps to ensure they are understood, accepted, and consistently applied by those responsible for carrying them out. Building shared understanding and commitment is essential for the long-term success of a lab—not only for data management, but for sustaining rigour and responsibility across *all* dimensions of the lab’s work. Shared norms and values are fundamental to good science in general, and to the development of legitimate, defensible and ethical practices in the production of scientific knowledge.

## Limitations and Future Work

While the results demonstrate the effectiveness of the DLC approach, they are drawn from laboratories that voluntarily chose to participate. This raises the possibility of self-selection bias: labs that opted into the study may already value strong data-management practices (though at least 2 PIs in the study indicated their interest in participating in the research precisely because they *didn’t* have a workable, or any, data management plan for their lab) and engage in effective, collaborative, inclusive communication. Such labs may have less room for improvement and therefore show smaller relative gains, meaning the findings could underestimate the potential impact for labs with weaker or less established practices or a less healthy culture/climate. Relatedly, however, these are also the types of labs most likely to join voluntary ethics focused programming if it were made available, and for that reason, the effect of the treatment on those *receiving* the treatment remains relevant even under conditions of self-selection.

We acknowledge the design limitations of a pre-post assessment in the absence of a comparison/control group. In this instance, we believe that a single-arm, pre-post design is defensible (and desirable, given the time and budget constraints of the current project) due to our prior results from using a variant of the proposed intervention with a more robust study design in the initial iREDS project, which included a comparison group study-arm, and which documented significant intervention effects for outcomes similar to those examined here. We believe that the difference in differences analyses performed in this study, which matched the RCT benchmarks in the prior study (Plemmons et al., 2020), provide a reasonable basis for interpreting the observed changes.

Future research should build on these findings by testing the DLC approach in a wider range of laboratories, employing recruitment strategies for those less likely to self-select into ethics-focused programming, as well as strategies designed to reach labs with more variable or less developed data-management practices; these approaches would help determine whether the intervention yields larger gains in those settings. In addition, longitudinal follow-up, beyond the 13–20 months found in this study, would help assess the durability of observed improvements and clarify possible mechanisms through which the DLC approach influences lab culture; this project wasn’t designed to explore those mechanisms We also did not design an exploration of the effects of this intervention on actual behaviour, though some of the responses in the follow up interviews indicate there may have been changes in that realm. It would be helpful to have an intentional approach to examining changes in behaviour over time. Finally, future work could explore how the DLC model might be adapted for more diverse research environments, or highly interdisciplinary teams, and how institutional policies, resources, or infrastructure can support broader dissemination and sustained implementation.

## Data Availability

The datasets generated and analyzed during the current study, and the code necessary to reproduce the results and figures, are available in the OSF repository. Plemmons, D., Esterling, K., & Gunsalus, C. K. (2025, October 12). Deliberative Lab Communication. Retrieved from https://osf.io/b6rm2/overview.

## Methods Appendix: Statistical Analysis

Our statistical tests rely on two designs: the within-subject design and the difference-in-differences design, with results reported in Figs. 1 and 2 of the main text.

**Within-Subject Design** The within-subjects design evaluates whether there is a *difference* between lab members’ pre-treatment $\:{O}_{i}^{0}$ and post-treatment $\:{O}_{i}^{1}$survey responses on average. The individual pretest and post-test responses each contain some degree of measurement error in addition to a systematic component that captures respondents’ latent opinion on that item at a given time. To address this, for the core analyses reported in Fig. 2 we create scales that measure latent concepts of interest. Mathematically, a “scale” is a weighted sum of individual survey items that are highly intercorrelated, where the weights are recovered from a statistical procedure that we describe below. Conceptually, one can think of these survey items as indicators that help to measure an underlying or latent variable that is of conceptual interest but that cannot be measured directly. For example, many of our concepts of interest focus on the nature of deliberative communication, lab climate, ethics beliefs, and scientific practice, and each of these concepts is difficult to measure accurately with ordinary survey responses. The virtue in focusing on scales is that the scales help to organize the data analysis on the larger concepts (or constructs) regarding within-subject changes over time at this more fundamental level.

We indicate a latent scale with $\:{\theta\:}_{i}^{}$ and to identify the scales we nest the set of questions for each scale within individual respondents. The likelihood for a single categorical outcome *k* is summarized in Eq. A.1.

A.1 $$\:{O}_{ik}\sim {OrderedLogit}\left({\lambda\:}_{k}{\theta}_{i}\right)$$

We estimate this model simultaneously for each of the survey items within a scale, and we model each of the six scales separately. In this equation, *i* indexes *N* participants and *k* indexes *K* questions for a given scale, and λ_k_ is a factor coefficient. To identify pre-post differences, we specify the linear equation:

A.2 $$\:{\theta}_{i}={\beta}_{0}+\:{\beta}_{1}{Post}_{i}+{\beta}_{2}{PI}_{i}+{\eta}_{i}$$

Where *Post* is an indicator of whether the response is on the post-test, and *PI* is an indicator if the respondent is the lab PI, and β_1_ is the pre-post within-subject difference. Finally, we note that the participants are each nested in a lab and so we model the dependence of responses within labs using a conditional autoregressive prior (Congdon 2003; Esterling et al. 2021) where $\:{\eta\:}_{i}$is the lab-level cluster random effect that accommodates spatial dependence of responses within a lab and

A.3 $$\begin{aligned}&{\eta}_{i}\sim\phi\:\big({\eta}_{i}^{*},1\big)\cr &{\eta}_{i}^{*}=\frac{\rho\:{\sum}_{j}\:{\eta}_{ij}}{{N}_{i}}\mathrm{,\:where\:}j\in\:\left\{i\mathrm{'s\:lab,\:}i\ne\:j\right\}\end{aligned}$$

and N_i_ is the number of lab members in *i*’s lab, not including respondent *i*. The ρ parameter captures the degree of autoregressive dependence of the latent scale responses within a lab and so $\:{\eta\:}_{i}$serves as a level-2 random effect that nets out lab-specific dependence from the treatment effect equation (Congdon 2003).

In the single equation models that we report in Figure 1 of the main text we use an identical approach to estimate the within-subject difference with nesting in labs, except the outcome of interest is an individual item instead of a scale. We estimate the single equation models using this same nesting approach, using a logit likelihood for the dichotomous responses and an ordered logit model for the model with an ordinal response.

**Difference-in-Differences Design** In the basic DID design, the researcher conducts a statistical test to see if there is a relationship between group exposure and the *difference* between the pre-treatment $\:{O}_{i}^{0}$ and post-treatment $\:{O}_{i}^{1}$ survey responses. The DID design proceeds by first taking the difference in the outcomes of the intervention and control groups in the time period after the intervention was administered, and then subtracting from that quantity the difference in outcomes between the two groups observed before the intervention. The DID design identifies the causal effect of this difference-in-differences using the “parallel path” assumption (Wing et al. 2018) that the intervention group would have had the same over-time trajectory in outcomes as the control group, had it not been exposed to the intervention.

Here we extend the DID design to accommodate comparisons across pre- and post-treatment scales. To derive the general model, initially assume a continuous, normally distributed opinion response at time *t*, $\:{O}_{i}^{t}$, and decompose the opinion response as

A.4 $$\:{O}_{i}^{t}={\beta}_{o}+{\lambda}_{1}^{t}{\theta}_{i}^{t}+{\varepsilon}_{i}^{t},\qquad\text{}t\in\left\{\mathrm{0,1}\right\}$$

where $\:{\theta\:}_{i}^{t}$ is the scale measuring each respondent’s latent opinion at time *t* and so measures the latent concept or construct of interest, $\:{\lambda\:}_{1}^{t}$ is a scaling structural parameter (sometimes referred to as a factor coefficient or discrimination parameter) and $\:{\epsilon\:}_{i}^{t}$ is the idiosyncratic component from measurement error that represents instability in the individual’s opinion response, all evaluated at time *t*; *t* = 0 is the pretest and *t* = 1 is the post-test. Similar to above, we identify$\:{\theta\:}_{i}^{t}$by nesting questions within participants.

To derive the DID statistical model for latent scales, we can take the difference in Eq. (A.4) between time *t* = 1 and *t* = 0,

A.5 $$\begin{aligned}&\text{}{O}_{i}^{1}={\beta}_{0}^{1}+{\lambda}_{1}^{1}{\theta}_{i}^{1}+{\varepsilon}_{i}^{1}\cr &{\beta}_{1}\left({O}_{i}^{0}={\beta}_{0}^{0}+{\lambda}_{1}^{0}{\theta}_{i}^{0}+{\varepsilon}_{i}^{0}\right)\end{aligned}$$

where *β*_*1*_ allows the underlying measurement scale to vary over time. Subtracting the second row of Eq. (A.5) from the first and rearranging yields,

A.6 $$\:{O}_{i}^{1}={\beta}_{0}+{\beta}_{1}{O}_{i}^{0}+\varDelta{\theta}_{i}+{\varepsilon}_{i}$$

where $\:{\beta\:}_{0}={\beta\:}_{0}^{1}-{\beta\:}_{1}{\beta\:}_{0}^{0}$ and $\:{\epsilon\:}_{i}={\epsilon\:}_{i}^{1}-{\beta\:}_{1}{\epsilon\:}_{i}^{0}$. With this derivation we have identified a new quantity of interest, $\:\varDelta\:{\theta\:}_{i}={\lambda\:}_{1}^{1}{\theta\:}_{i}^{1}-{\lambda\:}_{1}^{0}{\beta\:}_{1}{\theta\:}_{i}^{0}$ which is the change in the respondent’s pre- to post-discussion opinion in the scaled *latent opinion* space. This derivation allows us to isolate and measure the systematic component of opinion change using the latent scale, and we can test hypotheses regarding the differences in this latent scale between the experimental conditions, rather than relying on the noisily measured changes in the survey response itself.

In general, including an outcome response variable measured pretreatment, such as $\:{O}_{i}^{0}$, on the right hand side will lead to endogeneity bias since many of the individual-level determinants of an outcome in the pretreatment period also determine the outcome in the post-treatment period. In the statistical model below, we correct for this by conditioning on $\:{\theta\:}_{i}^{0}$ in the outcome equations. In essence, in this strategy we guard against endogeneity bias under the assumption that the latent preference scale is a strong predictor of both pre- and post-discussion responses, and that the remaining variation in $\:{O}_{i}^{0}$ and $\:{O}_{i}^{1}$ is conditionally independent.^13^ Thus, the equation we estimate is,

A.7 $$\:{O}_{i}^{1}={\beta}_{0}+{\beta}_{1}{O}_{i}^{0}+{\beta}_{2}{\theta}_{i}^{0}+\varDelta{\theta}_{i}+{\varepsilon}_{i}$$

We note too that modeling the $\:{\theta\:}_{i}^{0}$ and $\:\varDelta\:{\theta\:}_{i}$ parameters jointly with the structural parameters correctly propagates the uncertainty that comes from estimating these parameters through the statistical model. That is, the estimates of the structural parameters are the marginal distributions having integrated over the sample space underlying $\:{\theta\:}_{i}^{0}$ and $\:\varDelta\:{\theta\:}_{i}$, a technique known as the “method of composition” (Trier and Jackman 2008).

To identify the $\:{\theta\:}_{i}^{0}$ and $\:\varDelta\:{\theta\:}_{i}$ parameters, we nest the set of questions for each scale within individual respondents and set one factor coefficient to unity for each scale. The likelihood for a single categorical outcome is summarized in Eq. A.8, and the second row is a non-linear implementation of Eq. A.7.

A.8 $$\begin{aligned}&{O}_{ik}^{0}\sim{OrderedLogit}\left({\lambda}_{1k}{\theta}_{i}^{0}\right)\cr &{O}_{ik}^{1}\sim{OrderedLogit}\left({\beta}_{1k}{O}_{ik}^{0}+{\beta}_{2k}{\theta}_{i}^{0}+\varDelta{\theta}_{i}\right)\end{aligned}$$

We estimate this model simultaneously for each of the survey items within a scale, and we model each of the six scales separately. In this equation, *i* indexes *N* participants and *k* indexes *K* questions for a given scale.

Finally, we note that the participants are each nested in a lab and so we model the dependence of responses within labs using a conditional autoregressive prior (Congdon 2003). We define $\:\varDelta\:{\theta\:}_{i}$ as a normally-distributed random effect with conditional mean $\:\varDelta\:{\theta\:}_{i}^{*}$ and variance equal to one,^14^

A.6 $$\begin{aligned}&\varDelta{\theta}_{i}\sim\phi\left(\varDelta{\theta}_{i}^{*},1\right)\cr &\varDelta{\theta}_{i}^{*}={\alpha}_{1}{Z}_{i}+{\eta}_{i}\end{aligned}$$

where *Z*_*i*_ is the treatment assignment indicator. Since $\:\varDelta\:{\theta\:}_{i}$ is the difference in the pre- and post- treatment scale for a respondent, the structural parameter $\:{\alpha\:}_{i}$ is the difference-in-differences estimand for each scale. $\:{\eta\:}_{i}$is the lab-level cluster random effect that accommodates spatial dependence of responses within a lab and

A.7 $$\begin{aligned}&{\eta}_{i}\sim\phi\left({\eta}_{i}^{*},1\right)\cr &{\eta}_{i}^{*}=\frac{\rho \sum\limits_{j}{\eta}_{ij}}{{N}_{i}}\mathrm{,\:where\:}j\in\left\{i\mathrm{'s\:lab,\:}i\ne j\right\}\end{aligned}$$

and N_i_ is the number of lab members in i’s lab, not including the respondent. The *ρ* parameter captures the degree of autoregressive dependence of the latent scale responses within a lab and so $\:{\eta\:}_{i}$ serves as a level-2 random effect that nets out lab-specific dependence from the treatment effect equation (Congdon 2003).

### Estimation

We estimate all models in MultiBUGS version 2.0 using Bayesian MCMC methods (Goudie et al. 2020). All code to implement these models can be found at https://osf.io/b6rm2/overview. We assign Γ(0.1, 0.1) flat directional priors for all factor coefficient parameters (where the direction is assigned for identification), a log normal prior for the *ρ* parameter with log mean zero and log standard deviation 1, and flat N(0,1000) priors for all other parameters. We run the MCMC simulation from over dispersed initial values until the chains are stationary and then sample until the posterior distributions are smooth. The replication materials provide the traceplots and postestimation analysis for stationarity as well as all the sampled results we report.

### Missing Data Imputation

We impute the missing data as missing at random given the observed data and model-implied latent variables (Tanner and Wong 1987). The Bayesian model propagates the estimation uncertainty in the missing data imputations, and so the estimates of the structural parameters incorporate the additional uncertainty that is due to the missing data; that is, all parameters are marginalized over the full distribution of missing data.

## Footnotes

1 The original iREDS study was conducted at the University of California, Riverside, and The Scripps Research Institute in San Diego, California.

2 The total number of emails sent on our behalf was 1004, but of those, 36 were not eligible (retired, left the university, headed service organizations, did not have a lab).

3 Reasons for non-PI enrollment included: the PI was not at that time running a lab; the PI was director of a larger center or institute, without a lab; the PI didn’t respond to scheduling emails after initially expressing interest; the PI declined to participate or rescinded their enrollment for various reasons after the initial discussion with the PI of this study.

4 One lab was headed by two PIs.

5 There were 6 role conversions (e.g., post-doc to research scientist; undergraduate student to other/academic hourly) between the pre- and post-surveys; we report here on roles noted at the pre-survey.

6 In the analysis, we provide results from a difference-in-differences natural experimental design that makes use of the randomized control units from iREDS. The iREDS  study design, sample and sampling strategy are reported in Plemmons et al 2020. The number of iREDS control units is 89, with 27 participants completing the pretest but not the post-test, 12 completing the post-test but not the pretest, and 50 completing both pre- and post-test.

7 The National Center for Principled Leadership and Research Ethics (NCPRE), under the direction of CK Gunsalus, has developed resources based on these research findings, including orientation materials on the DLC and guidance for effectively managing these discussions, as well as offering suggestions for identifying possible facilitators and providing support to facilitators once identified (please see https://osf.io/b6rm2/overview for a list of and links to those resources). Resource development supported by NSF award # 2220604 in collaboration with this award.

8 The items for this sixth scale were only administered if the participant responded yes to the question asking if they knew their lab had a data management plan, and in the previous study, there were not enough yes responses to that question to justify a statistical test.

9 One of these 9 labs submitted two documents post-intervention: an unchanged from pre-intervention data management plan for a specific and on-going project, and a data management plan for a new project (and so there was no prior plan for comparison). Since we can’t use the 2nd plan for any comparison, only the first unchanged plan was included in the count with the 8 other unchanged plans, and the new plan not included in the analyses.

10 The DLC discussion for the 7 labs not included in the evaluation was facilitated by the post-doctoral scholar who conducted the evaluation interviews and so excluded.

11 There were an additional 2 interviews with PIs as pilot testing of the protocol, though we do include some of those responses here.

12 This project was not interested in data management practices per se, but only in whether the intervention led to increased understanding about/awareness of whatever plan the lab may have had, as well as the lab’s actual practices.

## References

[CR22] Congdon, P. (2003). Applied Bayesian modelling, Chapter 7. John Wiley & Sons, Ltd., Hoboken, NJ.

[CR1] Englund, T. (2006). Deliberative communication: A pragmatist proposal. *Journal of Curriculum Studies*, *38*, 503–520.

[CR23] Esterling, K. M., Fung, A., & Lee, T. (2021). When deliberation produces persuasion rather than polarization: Measuring and modeling small group dynamics in a field experiment. *British Journal of Political Science*, *51*, 686–704.

[CR2] Fryer-Edwards, K. (2002). Addressing the hidden curriculum in scientific research. *American Journal of Bioethics*, *2*, 58–59. 10.1162/152651602320957619 12762930

[CR3] Gastil, J. (2006). Communication as deliberation. In G. Shepherd, J. St. John, & T. Striphas (Eds.), *Communication as… Perspectives on theory* (pp. 164–173). Sage.

[CR24] Goudie, R. J. B., Turner, R. M., De Angelis, D., & Thomas, A. (2020). MultiBUGS: A parallel implementation of the BUGS modelling framework for faster Bayesian inference. *Journal of Statistical Software*, *95*(7). 10.18637/jss.v095.i07 10.18637/jss.v095.i07 PMC7116196 33071678

[CR4] Habermas, J. (1984). *The theory of communicative action, volume one: Reason and the rationalization of society* (T. McCarthy, Trans.). Beacon Press.

[CR5] Ingold, T. (1993). Technology, language, intelligence: A reconsideration of basic concepts. In K. R. Gibson, & T. Ingold (Eds.), *Tools, language and cognition in human evolution*. Cambridge University Press.

[CR6] Kalichman, M. (2014). A modest proposal to move RCR education out of the classroom and into research. *Journal of Microbiology & Biology Education*, *15*, 93–95. 25574254 10.1128/jmbe.v15i2.866 PMC4278527

[CR7] Lave, J. (1997). The culture of acquisition and the practice of understanding. In D. Kirshner, & J. A. Whitson (Eds.), *Situated cognition: Social, semiotic, and psychological perspectives* (pp. 63–82). Lawrence Erlbaum Associates.

[CR8] National Academies of Sciences, Engineering, and Medicine. (2017). *Fostering integrity in research*. National Academies. 29341557

[CR9] National Research Council & Institute of Medicine. (2002). *Integrity in scientific research: Creating an environment that promotes responsible conduct*. National Academies. 24967480

[CR10] Peiffer, A. M., Laurienti, P. J., & Hugenschmidt, C. E. (2008). Fostering a culture of responsible lab conduct. *Science*, *322*, 1186. 10.1126/science.322.5905.1186b PMC3967508 19023060

[CR11] Phillips, T., Nestor, F., Beach, G., & Heitman, E. (2018). America COMPETES at 5 years: An analysis of research-intensive universities’ RCR training plans. *Science and Engineering Ethics*, *24*, 227–249. 28299561 10.1007/s11948-017-9883-5 PMC5799314

[CR12] Pimple, K. D. (2013). Research conduct: Online integrity training falls short. *Nature*, *495*, 449. 10.1038/495449a 23538818

[CR14] Plemmons, D. K., & Kalichman, M. W. (2013). Reported goals of instructors of responsible conduct of research for teaching of skills. *Journal of Empirical Research on Human Research Ethics*, *8*, 95–103. 23651933 10.1525/jer.2013.8.2.95 PMC3705638

[CR15] Plemmons, D. K., & Kalichman, M. W. (2018). Mentoring for responsible research: The creation of a curriculum for faculty to teach RCR in the research environment. *Science and Engineering Ethics*, *24*, 207–226. 28281158 10.1007/s11948-017-9897-z

[CR13] Plemmons, D. K., Baranski, E. N., Harp, K., Lo, D. D., Soderberg, C. K., Errington, T. M., Nosek, B. A., & Esterling, K. M. (2020). A randomized trial of a lab-embedded discourse intervention to improve research ethics. *Proceedings of the National Academy of Sciences, 117*(3), 1389–1394. 10.1073/pnas.1917848117 10.1073/pnas.1917848117 PMC6983427 31919283

[CR16] Smale, N. A., Unsworth, K., Denyer, G., Magatova, E., & Barr, D. (2020). A review of the history, advocacy, and efficacy of data management plans. *International Journal of Digital Curation*, *15*(1), 30.

[CR17] Stahl, B. C. (2025). The ethics of data and its governance: A discourse theoretical approach. *Information*, *16*, 497.

[CR25] Tanner, M. A., & Wong, W. H. (1987). The calculation of posterior distributions by data augmentation. *Journal of the American Statistical Association*, *82*, 528–540.

[CR26] Trier, S., & Jackman, S. (2008). Democracy as a latent variable. *American Journal of Political Science*, *52*, 201–217

[CR18] Van Loon, J. E., Akers, K. G., Hudson, C., & Sarkozy, A. (2017). Quality evaluation of data management plans at a research university. *IFLA Journal*, *43*(1), 98–104. 10.1177/0340035216682041

[CR19] Whitbeck, C. (2001). Group mentoring to foster the responsible conduct of research. *Science and Engineering Ethics*, *7*, 541–558. 11697010 10.1007/s11948-001-0012-z

[CR20] Williams, M., Bagwell, J., & Nahm Zozus, M. (2017). Data management plans: The missing perspective. *Journal of Biomedical Informatics*, *71*, 130–142. 10.1016/j.jbi.2017.05.004 28499952 10.1016/j.jbi.2017.05.004 PMC6697079

[CR21] Wing, C., Simon, K., & Bello-Gomez, R. A. (2018). Designing difference-in-difference studies: Best practices for public health policy research. *Annual Review of Public Health*, *39*, 453–469. 10.1146/annurev-publhealth-040617-013507 29328877

